# Composite Grafting of a Distal Thumb Amputation: A Case Report and Review of Literature

**Published:** 2015-02-24

**Authors:** J. Choo, B. Sparks, M. Kasdan, B. Wilhelmi

**Affiliations:** ^a^Division of Plastic Surgery, University of Louisville; ^b^University of Louisville Medical School; ^c^Rex Robley Veterans Affairs Medical Center, Louisville, Ky

**Keywords:** thumb amputation, composite graft, finger amputation, replantation, distal thumb injuries

## Abstract

**Objective:** We report a case in which an avulsion-amputation of the thumb proximal to the lunula was repaired by reattaching the amputated segment as a composite graft. The graft demonstrated complete survival with only a minimal sacrifice in length. **Methods:** A 23-year-old man presented 4 hours after an avulsion injury of the thumb with associated distal and proximal phalanx fractures. The amputated segment included the sterile and germinal matrix. He underwent defatting and composite grafting of the amputated segment followed by K-wire fixation of his proximal phalanx fracture. **Results:** In his 1-week follow-up, the patient's composite graft—including his nail bed—demonstrated complete survival. At one month, the composite graft maintained stable soft tissue coverage and showed signs of nail plate regrowth. Four months after repair, he was able to return to light duty and was advanced to full duty within 5 months. He continued to report gradually improving hypersensitivity at the margins of the graft and stiffness of the interphalangeal joint. At five months he regained full mobility of his carpometacarpal joint. The range of motion of his interphalangeal and metacarpophalangeal joint were 0 to 10 degrees and 0 to 25 degrees, respectively. He was able to oppose his thumb to all 4 digits. Six months after repair, he demonstrated protective sensation of the tip of the thumb. **Conclusion:** Composite grafting of the thumb, even in less than ideal cases, can still provide useful length for function as a opposable post and can be considered in reconstruction of thumb amputations at or proximal to the lunula.

Fingertip amputations are the most common injuries to the upper limb. The specific goals of treatment after digital amputations can vary depending on the priorities and needs of the patient but can be broadly thought of in terms of restoring function, preserving sensation, and recreating a normal appearance. A critical component of thumb function is opposition, for which length, stability, and sensation are paramount.^1^

As with any fingertip injury, the age, comorbidities, occupation, and goals of the patient must all be considered in formulating a reconstructive plan for thumb amputations.^1^^,^^2^ Microsurgical replantation is the criterion standard of treatment in amputations proximal to the lunula. Unfortunately, many distal fingertip injuries are not amenable to replantation due to the location or mechanism of injury or lack of surgical expertise. Alternative management strategies range from conservative measures such as allowing healing by secondary intention^3^ and skin grafts^4^ to various local^5^^-^7 and locoregional flaps.^8^^,^^9^ Disadvantages of these flaps range from stiffness related to prolonged immobility and donor site morbidity.^10^ Revision amputation is another common treatment modality that is often indicated if the patient desires a speedy recovery and quicker return to work but may result in the sacrifice of further length to obtain the necessary soft tissue coverage.

Composite grafting of the amputated thumb is a simple and cost-effective technique that allows for the preservation of length while avoiding the donor site morbidity of locoregional flaps. Douglas reported the successful use of composite grafts as early as 1959,^2^ the technique has been discouraged in all but the youngest children due to historically poor outcomes.^11^ However, several more recent studies have reported acceptable survival rates of composite grafting in the appropriately selected adult patients.^11^^-^13

The authors contend that composite grafting has a place in the reconstructive algorithm of distal thumb amputations. In fact, as the following case report illustrates, composite grafting may be an alternative for reconstruction that avoids many of the drawbacks of other methods.

## CASE REPORT

**Figure 1:**
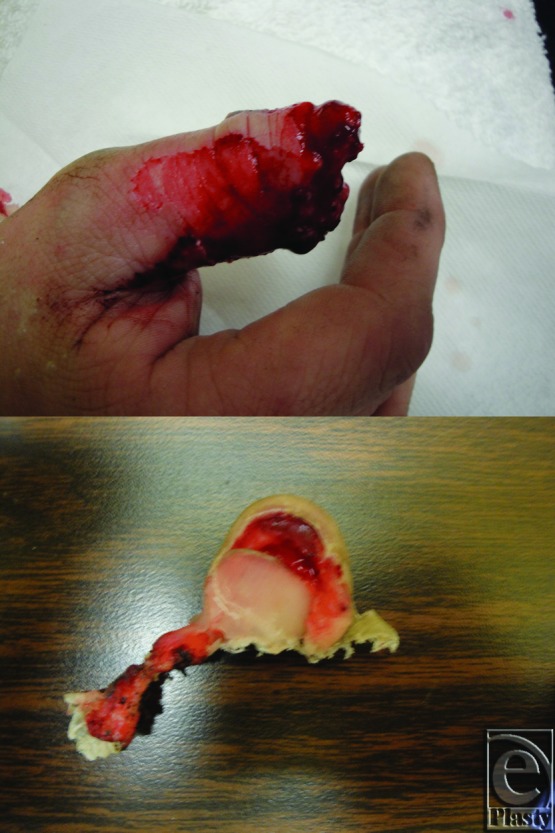
Amputation with significant soft tissue degloving immediately distal to the IP joint. The nature of the injury precluded microvascular repair, and an amputation-revision would have necessitated removing the IP joint (*above*). The amputated segment including nail plate, underlying nail bed, and pulp (*below*).

Our patient is a healthy 23-year-old man, who presented 4 hours after sustaining an avulsion/degloving injury proximal to the lunula of the left thumb. The avulsed segment of thumb included the nail bed, both sterile and germinal matrix, hyponychium as well as approximately 3 to 4 mm of distal phalanx (DP) (Fig 1). The remaining DP was partially denuded but intact. Rather than a simple transverse amputation, the amputated segment had a “cap” configuration in relation to the remaining stump. Intraoperative fluoroscopy revealed an associated oblique fracture of the proximal phalanx. Intraoperatively, no suitable vessels were available for microsurgical repair. The patient also did not want a procedure that would involve an additional scar on his hand. In addition, while the injury level was distal to the IP joint, the authors felt that a revision amputation would require an unacceptable loss of length to obtain the necessary soft tissue coverage.

**Figure 2:**
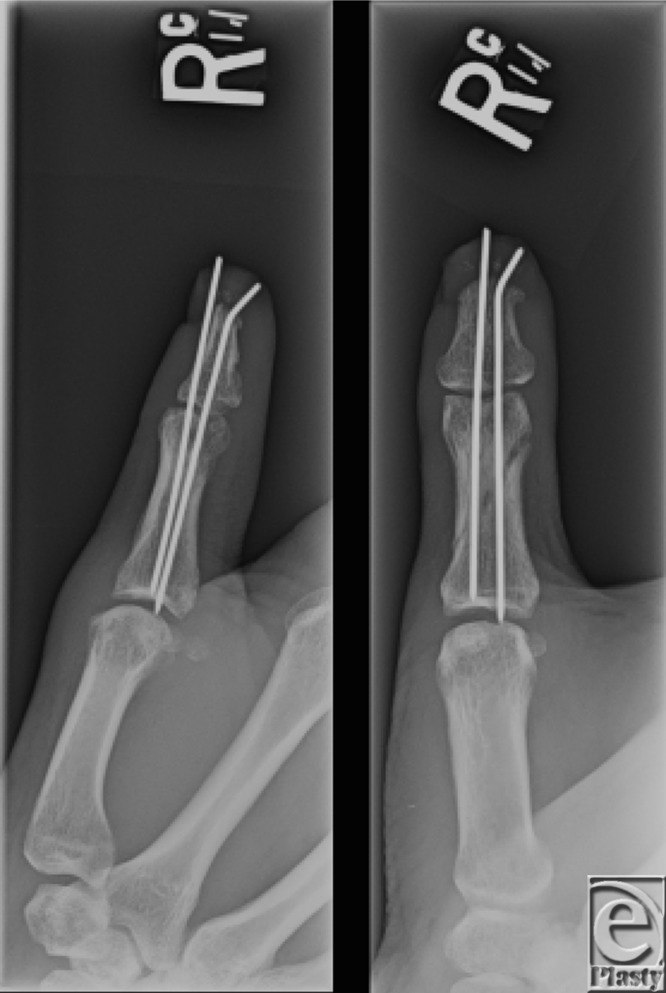
Intraoperative X-ray showing approximately 2- to 3-mm loss of distal phalanx, and adequate reduction of the proximal phalanx fracture after pinning.

The patient opted to undergo composite grafting of his distal thumb. Careful debridement of nonviable tissue was performed with sparing use of electrocautery. A closed reduction and pinning of the proximal phalanx fracture was accomplished under fluoroscopic guidance using two 0.045" K wires (Fig 2).

The composite graft was debrided and excess subcutaneous fat was excised as in the manner of a thick full-thickness skin graft. Two K wires were passed retrograde through the composite graft, which was then secured circumferentially using multiple nonabsorbable sutures in an interrupted fashion. The wound was then dressed and the thumb placed in a spica splint.

**Figure 3:**
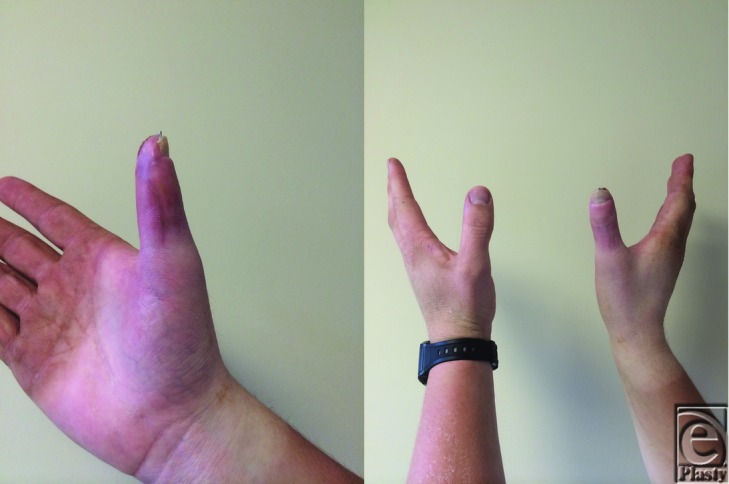
One-month postoperative result showing survival of composite graft with stable soft tissue coverage (*left*). A comparison of the repaired and noninjured thumb (*right*). A slight loss of length is noted, which is attributed to the loss of length of the distal phalanx and the fact that, at 1 month, the nail plate had not regrown fully.

Postoperatively, the patient was discharged home after his procedure. In his 1-week follow-up, the patient's composite graft—including his nail bed—demonstrated complete survival. At one month, the composite graft maintained stable soft tissue coverage and showed signs of nail plate regrowth (Fig 3). Four months after repair, he was able to return to light duty and was advanced to full duty within 5 months. He continued to report gradually improving hypersensitivity in the graft and stiffness of the interphalangeal (IP) joint. Hypersensitivity was self-reported as pain and numbness of the thumb tip in cold weather or grasping cold objects. At 5 months, he regained full mobility of his carpometacarpal joint. The range of motion of his IP and metacarpophalangeal (MCP) joint were 0 to 10 degrees and 0 to 25 degrees, respectively. He was able oppose his thumb to all 4 digits. Six months after repair, he demonstrated protective sensation of the tip of the thumb with static 2-point discrimination of 15 mm. In comparison, his left thumb demonstrated 10 degrees of extension to 45 degrees of flexion at the MCP joint and 10 degrees of extension to 75 degrees of flexion at the IP joint. His static 2-point discrimination in the uninjured thumb tip was 7 mm.

## DISCUSSION

Fingertip amputations are the most common injuries to the upper limb. Several classification systems exist for anatomical localization of fingertip injuries. Two of the more commonly referenced are by Allen and Hirase. Allen's classification^3^ can be utilized preoperatively as it employs gross landmarks to describe the level of amputation. According to the Allen classification, type I injuries involve only the pulp of the fingertip; type II include loss of both pulp and nail; type III injuries include partial loss of the terminal phalanx plus corresponding pulp and nail; and type IV are proximal to the lunula and involve the germinal matrix in addition to the terminal phalanx, nail, and pulp.^3^

In the thumb, it can be also useful to assess amputations in terms of functional level.^1^^,^^14^ Amputations of the thumb distal to the IP joint, which is our exclusive focus here, preserve enough thumb length to permit pinch and grasp, and are therefore grouped in 1 functional category. In these situations, the main priority of reconstruction tends to focus on providing durable and sensate skin rather than maximizing length.^14^ Injuries proximal to the IP joint have a higher likelihood of requiring some form of lengthening.^14^

Microsurgical replantation, when possible, is the preferred method of repair for distal thumb amputations. It has the advantage of restoring length and normal appearance of the injured digit while avoiding donor site morbidity. Successful revascularization of the replanted thumb can be expected in 80% of cases,^15^ but the true test of success is thumb function, which is predicated on sensation.^15^ Depending on the level and type (crush vs laceration), 2-point discrimination ranges from 8 mm to 15 mm.^15^ Nevertheless, even a thumb with poor mobility and sensation can be useful as a post for opposition.^15^^-^17 Consequently, vascular compromise of thumb replants/revascularization, which generally leads to fingers that are atrophic and have poor sensation and mobility, can still yield an acceptable functional result in the thumb.^15^ A composite graft can be thought of as the ultimate example of a replant with vascular compromise. While atrophy of the reattached segment diminished sensation and mobility may occur, many of the benefits of thumb replantation are retained.

In our case, proximal thumb amputation reconstructed with composite grafting ultimately led to a stable skin cover and a thumb that was usable as a post for opposition and demonstrated protective sensation at the tip. Stiffness and loss of range of motion of the IP and MCP joints could not be solely attributed to the method of repair as the patient required closed reduction and percutaneous pinning of the associated proximal phalanx fracture.

Amputation is a straightforward option if the patient desires to return to work quickly, provided that length beyond the IP joint can be maintained to permit opposition of the thumb. For injuries occurring at the proximal to the lunula or accompanied by unfavorable soft tissue loss, an amputation-revision may not be possible without an unacceptable loss of thumb length. In these situations, local and locoregional flaps such as the Moberg flap, cross finger flap, and first dorsal metacarpal artery flap are commonly used. All have their utility as well as drawbacks, including stiffness in the case of Moberg flaps and additional donor site morbidity with cross finger and first dorsal metacarpal artery flaps, as well as sensory relearning.

The Hirase classification system^18^ proposed that the level of injury in relation to the level of anastomosis of the digital artery determined the surgical method of reattachment. If, for example, the injury is too distal to permit an arterial anastomosis, it is treated with a nonvascularized composite graft and classified accordingly as a DP-I injury. If the level of amputation is at the distal arch of the digital artery or one of its branches and an arterial anastomosis is possible, it is classified as a DP-II injury. Any injury proximal to the distal arch of the digital artery is classified as a DP-III injury and necessitates an arterial and venous anastomosis for survival. An important distinction of Hirase's classification system is that the level of injury usually cannot be determined preoperatively. Indeed, the author did not attempt to define external landmarks that correspond to the type of vascular repair required for reattachment.^18^

While such classification systems have sought to establish when a composite graft might be indicated, the established consensus on composite grafting of distal fingertip injuries is that there are very few indications, if any, in which composite grafts are the recommended treatment modality for DP amputations. The risks of using composite grafts for more proximal amputations would be expected to be even more prohibitive. The rationale for such a view is not hard to comprehend, although the evidence on which this is based is difficult to find and inconsistent. For example, although countless reports state that composite grafts are controversial and should not be attempted in adults due to low rates of success, only one study was found which actually recommended that the technique be limited to children and young adults.^19^ In this article, no evidence was presented to support this recommendation.

As Hirase's classification scheme alludes to, a few studies have argued for the role of composite grafting in amputations distal to the termination of the digital artery.^18^^,^^20^ In fact, many of the recent reports demonstrate acceptable survival rates in adults with the implementation of new surgical techniques or postoperative care strategies to aid in the success of graft survival.^10^^-^13^,^^18^^-^21

There are numerous well-known factors that have demonstrated poor survival rates of composite grafts including smoking, prolonged time (>5 hours) to replantation,^12^ larger amount of tissue amputated based on the anatomic location of injury,^3^^,^^21^ and crush injuries.^22^ Patient age is repeatedly mentioned^19^^,^^21^^,^^22^ as a prognostic factor in graft survival; however disagreement exists as to what the “cut-off” age should be, if any.^10^^,^^13^ Some sources state that composite grafts should not be attempted in patients older than 6 years, while recent studies show promising results in adult patients. Interestingly, Moiemen and Elliot^11^ found that the level of the amputation did not significantly affect the graft as one would expect; amputations at the level of the lunula did just as well as amputations that were more distal.

Several surgical techniques have been created in efforts to maximize composite graft take. The “cap” technique described by Rose^23^ enhances graft survival by increasing the surface area between the graft and the stump. This principle was reinforced by Uysal with ostectomy, defatting, and stump deepithelization.^21^ The use of subcutaneous pockets has also proven to aid in graft survival.^24^^-^26 In addition to surgical techniques, numerous postoperative strategies have been employed including digital cooling with foil and ice,^27^ the use of hyperbaric oxygen,^28^^,^^29^ and pharmacologic agents such as prostaglandin-E1.^18^^,^^30^^,^^31^ Many of these studies start by stating that composite grafting should not be attempted in adults; however, they repeatedly show high rates of success.

While it seems that an argument can be made for the survival of composite grafts, the true test of success is useful function and sensation. While an outright comparison of sensibility to locoregional flaps has not been done, composite grafts have been shown to compare favorably to common locoregional flaps with regard to 2-point sensitivity.^9^^,^^10^ It would be interesting to study how methods to increase graft survival such as defatting affect outcomes in regard to sensibility, cold intolerance, and protective sensation.

Composite grafting can demonstrate long-term viability. The main issue in determining their usefulness is the stability of soft tissue coverage and usable sensitivity. In our case report, composite grafting led overall maintenance of thumb length, stable soft tissue coverage, and function as a post for opposition. Despite its clear limitation and many unanswered questions, composite grafting may be considered for amputations that are in the proximal end of the DP, as in our case. With the appropriate technique, composite grafting has acceptable survival rates while maintaining length and preserving the nail plate, and provides durable soft tissue coverage while avoiding the donor site morbidity of locoregional flaps.
